# Development and validation of a novel lipid metabolism-related gene prognostic signature and candidate drugs for patients with bladder cancer

**DOI:** 10.1186/s12944-021-01554-1

**Published:** 2021-10-27

**Authors:** Ke Zhu, Liu Xiaoqiang, Wen Deng, Gongxian Wang, Bin Fu

**Affiliations:** 1 Present Address: Department of Urology, The First Affiliated Hospital of Nanchang University, 17 Yongwaizheng Street, Jiangxi 330006 Nanchang, People’s Republic of China; 2 Jiangxi Institute of Urology, Jiangxi 330006 Nanchang, People’s Republic of China

**Keywords:** Bladder cancer, Lipid metabolism, Signature, TCGA, GEO, Biomarker, Prognosis, Immune

## Abstract

**Background:**

Bladder cancer (BLCA) is a common cancer associated with an unfavorable prognosis. Increasing numbers of studies have demonstrated that lipid metabolism affects the progression and treatment of tumors. Therefore, this study aimed to explore the function and prognostic value of lipid metabolism-related genes in patients with bladder cancer.

**Methods:**

Lipid metabolism-related genes (LRGs) were acquired from the Molecular Signature Database (MSigDB). LRG mRNA expression and patient clinical data were obtained from The Cancer Genome Atlas (TCGA) and Gene Expression Omnibus (GEO) datasets. Cox regression analysis and least absolute shrinkage and selection operator (LASSO) regression analysis was used to construct a signature for predicting overall survival of patients with BLCA. Kaplan-Meier analysis was performed to assess prognosis. The connectivity Map (CMAP) database was used to identify small molecule drugs for treatment. A nomogram was constructed and assessed by combining the signature and other clinical factors. The CIBERSORT, MCPcounter, QUANTISEQ, XCELL, CIBERSORT-ABS, TIMER and EPIC algorithms were used to analyze the immunological characteristics.

**Results:**

An 11-LRG signature was successfully constructed and validated to predict the prognosis of BLCA patients. Furthermore, we also found that the 11-gene signature was an independent hazardous factor. Functional analysis suggested that the LRGs were closely related to the PPAR signaling pathway, fatty acid metabolism and AMPK signaling pathway. The prognostic model was closely related to immune cell infiltration. Moreover, the expression of key immune checkpoint genes (PD1, CTLA4, PD-L1, LAG3, and HAVCR2) was higher in patients in the high-risk group than in those in the low-risk group. The prognostic signature based on 11-LRGs exhibited better performance in predicting overall survival than conventional clinical characteristics. Five small molecule drugs could be candidate drug treatments for BLCA patients based on the CMAP dataset.

**Conclusions:**

In conclusion, the current study identified a reliable signature based on 11-LRGs for predicting the prognosis and response to immunotherapy in patients with BLCA. Five small molecule drugs were identified for the treatments of BLCA patients.

**Supplementary Information:**

The online version contains supplementary material available at 10.1186/s12944-021-01554-1.

## Introduction

Bladder cancer (BLCA) is one of the most common malignancies of the genitourinary system, and it is also the 12th most common cancer worldwide [1]. The incident risk of BLCA is closely correlated with smoking [2]. Based on muscular invasion, BLCA can be classified into two types: non-muscle-invasive BLCA and muscle-invasive BLCA. The former is generally treated with transurethral bladder tumor resection (TURBT) and regular intravesical instillation. However, this type of BLCA easily recurs and progresses. The latter usually requires radical cystectomy and urinary diversion, even chemotherapy, immunotherapy, and targeted therapies. Moreover, patient prognosis may remain unfavorable.Therefore, it is essential to identify early diagnostic and prognostic biomarkers to improve the curative effects of BLCA.

Emerging evidence has confirmed that aberrant metabolic reprogramming, especially glycolysis [3, 4], mitochondrial oxidative phosphorylation [5, 6], cholesterol metabolic pathways [7], and fatty acid metabolism [8], contributes to the occurrence of many diseases, including cancers and inflammation [9]. Lipids are not only one of the three nutrient types necessary for normal cell growth but also components of cell membranes, and these roles determine the significant effect of lipids on cell growth and homeostasis. Recently, lipid metabolism disorder has been regarded as one of the most significant metabolic hallmarks of tumor cells [10, 11]. Lipids not only provide nutrition for the malignant proliferation of tumor cells but also can favor tumor cells adaptation to microenvironmental changes. Bladder carcinogenesis is associated with alterations in lipid metabolism [12–14]. Overexpression of fatty acid synthase (FASN) has been found to be negatively correlated with OS and recurrence [15]. Furthermore, silencing FASN expression significantly suppressed the proliferation and invasion of BLCA cells through the AKT/mTOR signaling pathway [16]. FASN might contribute to chemotherapy resistance [17].

In the present study, the expression and potential functions of lipid metabolism-related genes in BLCA systematically analyzed by a series of bioinformatic methods. Then, five small molecule compounds that target lipid metabolism related genes were identified for BLCA treatment. Finally, a prognostic signature based on eleven lipid metabolism-related genes that can accurately predict BLCA patient prognosis was constructed and validated. Furthermore, the prognostic signature was an independent prognostic indicator and that was correlated with immune cell infiltration. All these results indicated that lipid metabolism may be a promising treatment direction for BLCA.

## Materials and methods

### TCGA-BLCA cohort and GEO cohort

The level-three transcriptome RNA sequencing data and the corresponding clinicopathological characteristics of bladder cancer patients were downloaded from The Cancer Genome Atlas (TCGA) data portal (https://gdc-portal.nci.nih.gov/). Moreover, GSE13507 was obtained from the Illumina Human-6 v2.0 Expression BeadChip platform in Gene Expression Omnibus (GEO) database (https://www.ncbi.nlm.nih.gov/geo/) and used as a validation set.

### Lipid metabolism gene set

Four lipid metabolism datasets (Reactome metabolism of lipids, Reactome phospholipid metabolism, Hallmark fatty acid metabolism, and Kyoto Encyclopedia of Genes and Genomes (KEGG) glycerophospholipid metabolism) were acquired from the Molecular Signature Database v7.1 (MSigDB; https://www.gsea-msigdb.org/gsea/msigdb).

### Identification of differentially expressed lipid metabolism-related genes

The Limma package of R (version R 3.6.1, https://bioconductor.org/packages/release/bioc/) was used to screen the differentially expressed lipid metabolism-related genes (DELRGs) between the BLCA and normal samples. A false discovery rate (FDR) < 0.05 and |log2-fold change (FC)| > 1 were set as the cutoff criteria.

### Enrichment analysis of DELRGs

To further investigate the potential molecular mechanisms in which the DELRGs were involved, Gene Ontology (GO) and pathway enrichment analyses of the DELRGs were performed with the clusterProfiler R package. *P* and FDR values < 0.05 were considered statistically significant.

### Protein-protein interaction (PPI) network construction

DELRGs were submitted to the STRING database (http://www.string-db.org/, version 11.0) to acquire PPI information and visualized by Cytoscape (version 3.8.2).

### Identification of potential small molecule drugs

The connectivity Map (CMAP) database (http://www.broadinstitute.org) was used to predict potential drugs that may reverse or induce the biological states of BLCA based on the DELRGs. The DELRGs was submitted to the CMAP database to search small molecular drugs that could be used for BLCA treatment. The enrichment scores ranged from − 1 to 1. A negative score suggested that the drug could be beneficial for BLCA treatment.

### Construction and validation of LRG prognostic signature

Univariate Cox regression was used to identify the prognostic value of LRGs in BLCA. Then, LASSO regression analysis was conducted to select potential risk genes and eliminate genes that would overfit the model. Finally, multivariate Cox regression analysis was performed to establish an optimized risk score model. Risk scores was calculated by the following formula:

$$\mathrm{Risk}\;\mathrm{score}\;=\;\left(\mathrm{Coef}1\ast\mathrm{expression}\;\mathrm{mRNA}1\right)\;+\;\left(\mathrm{Coef}2\ast\mathrm{expression}\;\mathrm{RNA}2\right)\;+\;\left(\mathrm{Coef}\;\mathrm n\;\ast\;\mathrm{expression}\;\mathrm{mRNA}\;\mathrm n\right)$$

where Coef was Cox regression model coefficient of relevant mRNA. Patients with ccRCC were classified into two groups (low-risk groups and high-risk groups) on the basis of median risk score. Kaplan-Meier curves were plotted to evaluate the significant difference in survival outcomes between the high risk and low risk groups. Principal component analysis (PCA) and t-distributed stochastic neighbor embedding (t-SNE) were used to analyze the dimensionality reduction. The survival ROC package was used to construct the receiver operating characteristic curve (ROC). The prognostic model was externally validated using the GEO dataset to test its stability.

### Construction of a nomogram

Univariate and multivariate Cox regression analyses were conducted to determine if the predictive effect of the LRG prognostic signature was independent of clinical variables. A nomogram survival model was established using the R package rms based on the independent prognosis-associated LRGs to predict the survival rate of BLCA patients at 3 and 5 years. The nomogram and calibration curve were plotted with the “rms” R package. The accuracy of the nomogram was estimated by calculating the consistency index between the actual observation frequency and the predicted probability. A calibration curve was utilized to visualize the performance of the nomogram.

### Immune cells infiltration

The CIBERSORT, MCPcounter, QUANTISEQ, XCELL, CIBERSORT-ABS, TIMER and EPIC algorithms were used to analyze the immunological characteristics of the high-risk groups and low-risk groups. To predict the effect of immune checkpoint blockade therapy, we also explored the expression of key immune checkpoint genes including PDCD1, LAG3, HAVCR2, PD-L1 and CTLA4 in the groups.

### Validation of protein expressions of 11 LRGs

The Human Protein Atlas (HPA, https://www.proteinatlas.org/) database was conducted to validate the protein expression of 11 LRGs between BLCA tissues and normal bladder tissues via using immunohistochemistry (IHC) from HPA database.

### Statistical analysis

All the statistical analyses were conducted with R software (Version 3.5.0). *P* < 0.05 was served as the cutoff criterion.

## Results

### Identification of DELRGs in BLCA

**Fig. 1:**
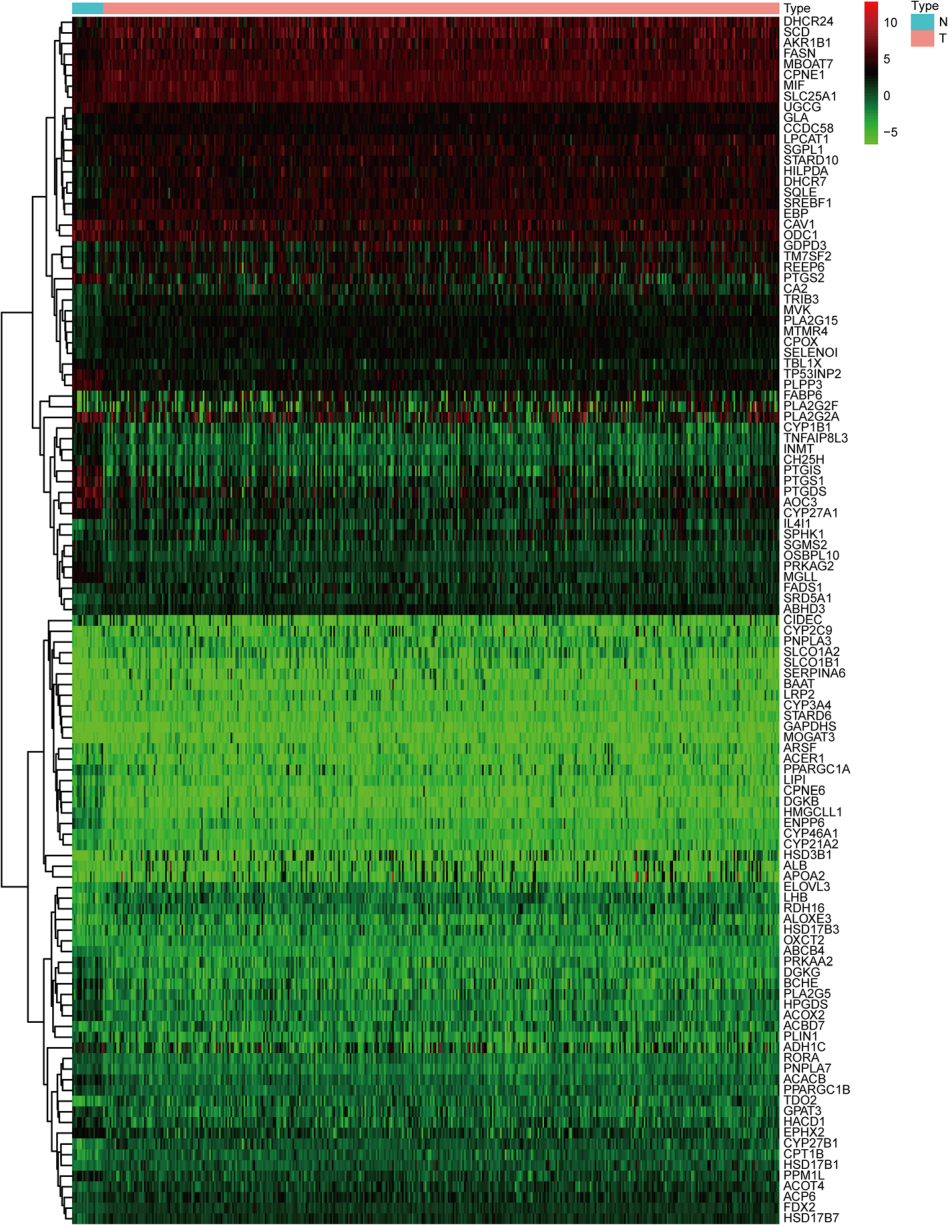
Heatmap showed differentially expressed LRGs

The RNA-seq data of 857 LRGs between BLCA tissues (*n* = 414) and normal bladder tissues (*n* = 19) were acquired from TCGA dataset. 113 DELRGs were identified with FDR < 0.05 and | log2 FC| >1 as the screening criteria, including 49 downregulated and 64 upregulated genes. The heatmap of the DELRGs between the BLCA tissues and normal bladder tissues was displayed in Fig. 1.

### Functional enrichment analysis of DELRGs

**Fig. 2:**
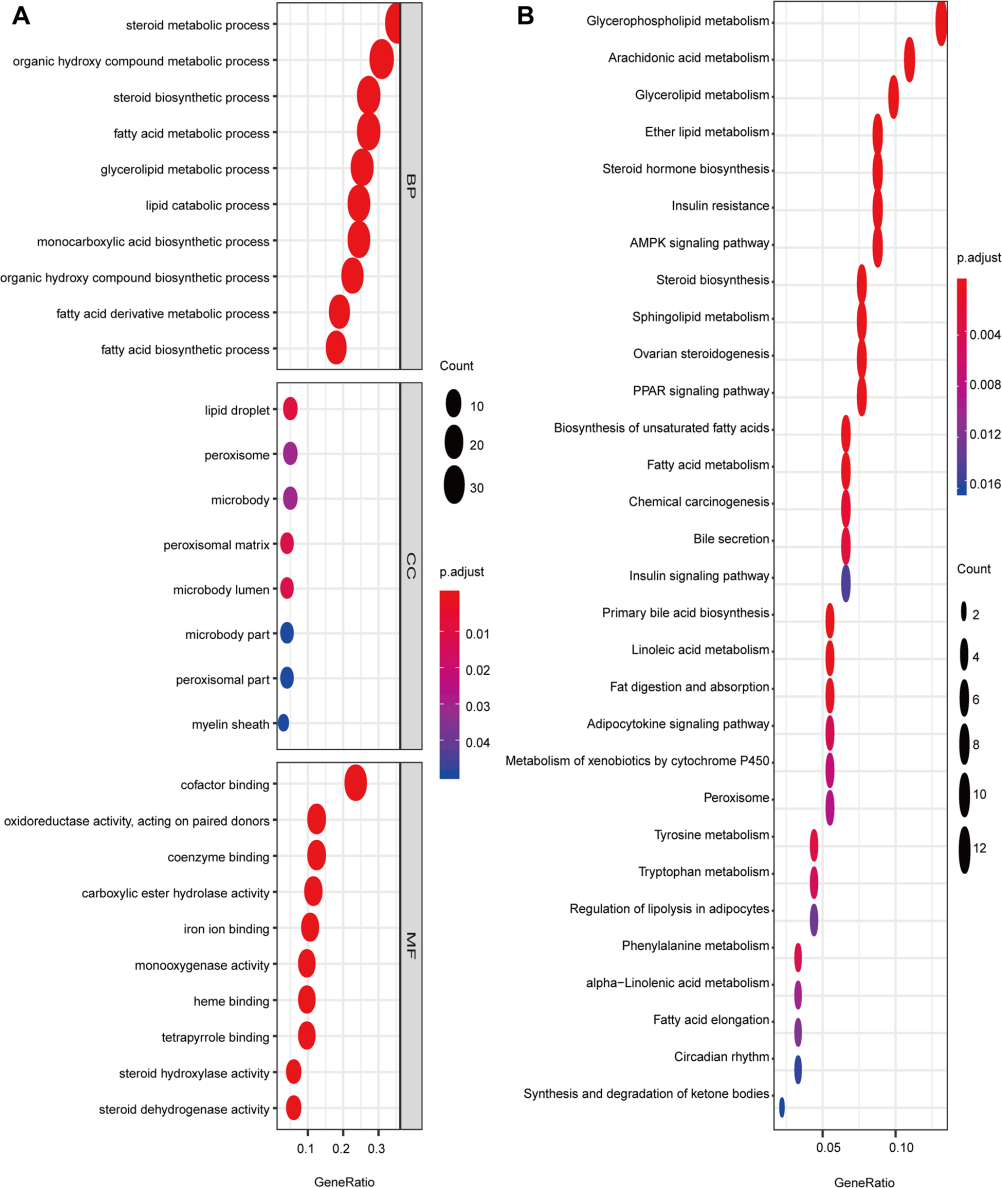
Enrichment analysis of differentially expressed LRGs. **A** GO analysis including biological process, cellular component and molecular function; **B** KEGG analysis

To further clarify the potential mechanisms of DELRGs, functional enrichment analysis was performed with the 113 DELRGs. In the biological processes (Fig. 2A), the DELRGs were mainly enriched in steroid metabolic process, fatty acid metabolic process, lipid catabolic process, steroid biosynthetic process, and fatty acid derivative metabolic process. In the cellular components (Fig. 2A), the DELRGs were mainly enriched in lipid droplet, peroxisomal matrix, myelin sheath, microbody lumen, peroxisome, and microbody. In the molecular functions (Fig. 2A), the DELRGs were mainly enriched in cofactor binding, monooxygenase activity, iron ion binding, phospholipase A2 activity, and NADP binding. In the KEGG pathways, the results showed that the DELRGs were mainly enriched in glycerophospholipid metabolism, PPAR signaling pathway, fatty acid metabolism, and AMPK signaling pathway (Fig. 2B). All these results indicated that lipid metabolism might be implicated in the development of BLCA.

### PPI network

**Fig. 3:**
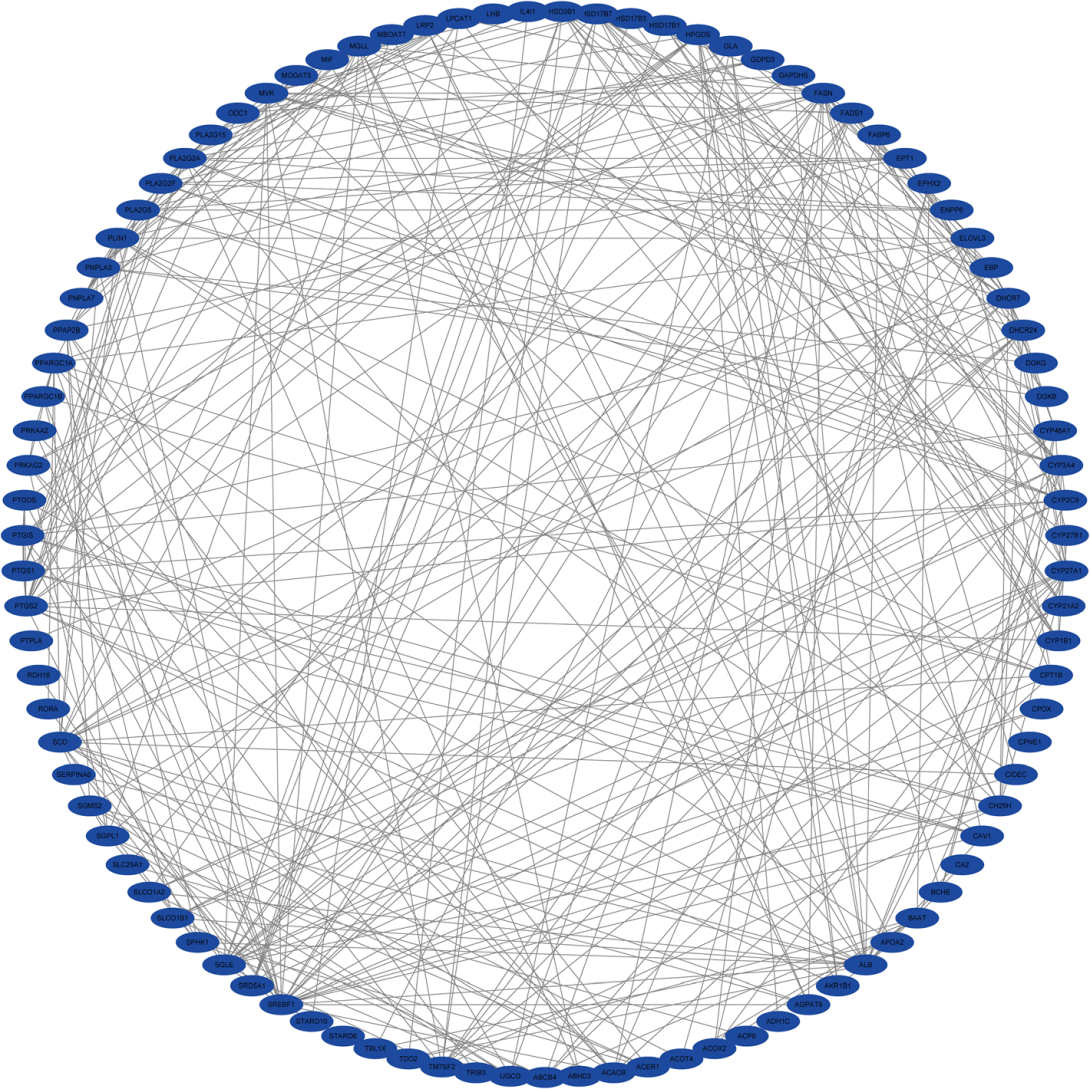
Protein-protein interaction (PPI) network of differentially expressed LRGs

To investigate the association of the DELRGs, a PPI network of the DELRGs was established comprising 95 nodes and 350 edges and visualized via Cytoscape (Fig. 3).

### Small molecular drugs

To identify candidate small molecular drugs for treating BLCA, all the DELRGs were divided into upregulated and downregulated groups, which were uploaded to the CMAP database. Five small molecular drugs with anticancer effects on BLCA progression were identified (enrichment score < 0) with *P* < 0.01 and *n* > 2 as the screening criteria. The 5 small molecule drugs were flurbiprofen, meclizine, alfuzosin, ethotoin, and fenoprofen (Table 1).

**Table 1:** The 5 small molecule drugs of CMP dataset

Names of drugs	Enrichment	*p *value	n	Percent non-null
flurbiprofen	-0.947	0.00026	3	100%
meclozine	-0.911	0.00124	3	100%
alfuzosin	-0.894	0.00226	3	100%
ethotoin	-0.893	0.00234	3	100%
fenoprofen	-0.848	0.00699	3	100%

### Construction of LRGs signature for predicting OS

**Fig. 4:**
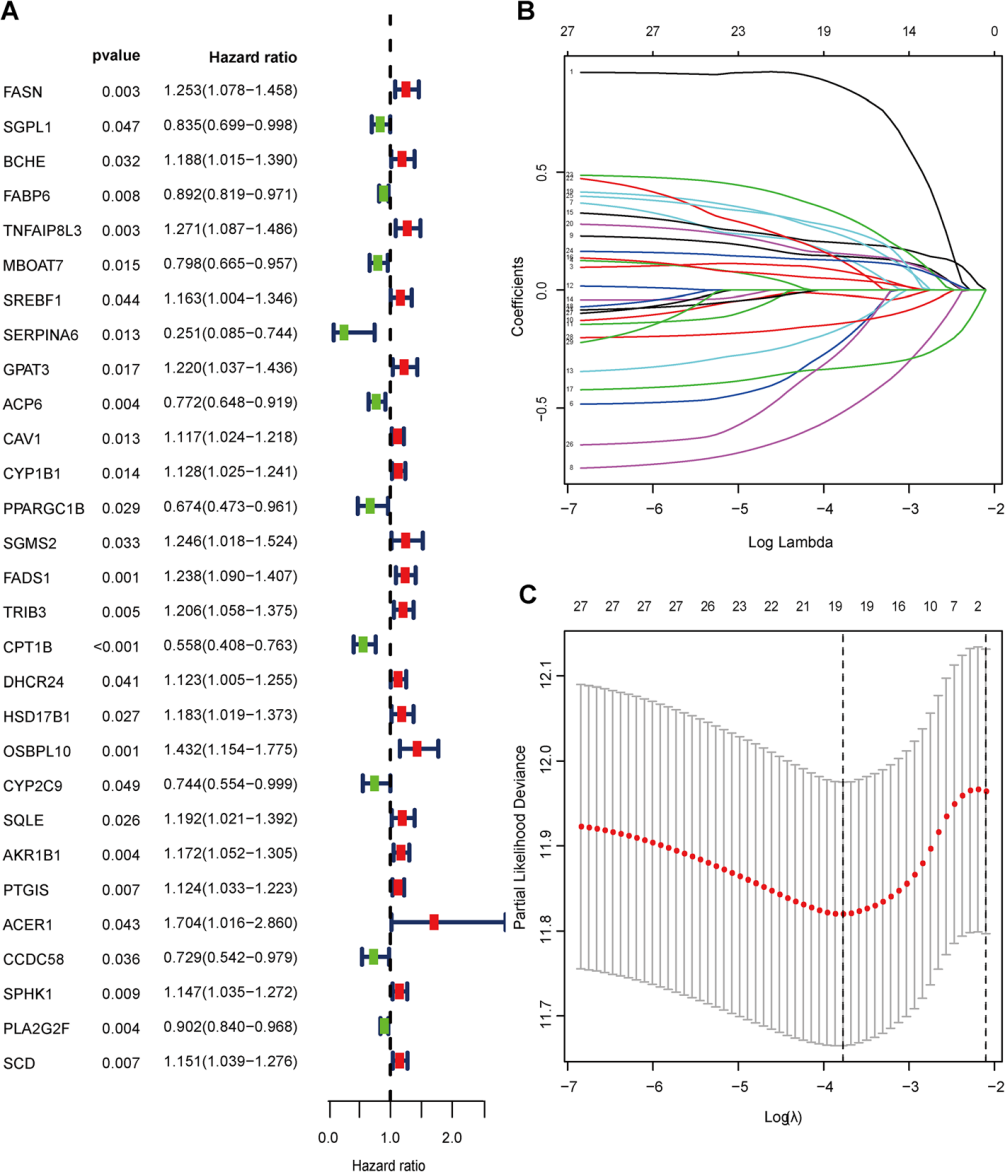
Identification of DELRGs closely related to prognosis. **A** Identification of differentially expressed LRGs by univariate Cox regression analysis. **B** The coefficient profile of 19 prognostic genes by Lasso regression analysis. **C** Tenfold cross-validation for tuning parameter selection in the LASSO analysis

Based on the 113 DELRGs, Cox and LASSO regression analyses were conducted to select prognostic genes in the TCGA dataset. First, twenty-nine DELRGs were associated with the prognosis of BLCA patients according to univariate Cox regression analysis (Fig. 4A). Then, to ensure the stability and feasibility of clinical prognosis based on these 29 genes, we obtained 19 DELRGs associated with the prognosis of BLCA patients by LASSO analysis (Fig. 4B and C). Finally, after multivariate Cox regression analysis, 11 genes, including FASN, MBOAT7, SERPINA6, PPARGC1B, FADS1, CPT1B, HSD17B1, OSBPL10, AKR1B1, CCDC58, and PLA2G2F, were identified and used to construct a prognostic signature for OS (Table 2). We developed an 11 gene signature-based risk score based on their cox coefficient as follows:

**Table.2:** The multivariate Cox regression analysis of11 prognostic genes in bladder cancer

Gene names	HR	95% CI	Coef	*p*-value
FASN	1.314	1.114-1.549	0.2728	0.001
MBOA7	0.811	0.661-0.994	-0.2101	0.043
SERPINA6	0.408	0.146-1.141	0.8956	0.088
PPARGC1B	0.701	0.486-1.01	-0.3559	0.057
FADS1	1.122	0.962-1.31	0.1154	0.143
HSD17B1	1.206	1.027-1.416	0.1872	0.022
AKR1B1	1.123	0.997-1.264	0.1156	0.057
PLA2G2F	0.938	0.861-1.022	0.064	0.142
CCDC58	0.784	0.563-1.091	-0.2434	0.149
CPT1B	0.752	0.532-1.064	-0.2844	0.107
OSBPL10	1.213	0.946-1.555	0.193	0.128

$$\mathrm{Risk}\;\mathrm{score}\;=\;(0.2728\;\times\;\mathrm{FASN}\;\mathrm{expression})\;+(-0.2101\;\times\;\mathrm{MBOAT}7\;\mathrm{expression})\;+\;(0.8956\;\times\;\mathrm{SERPINA}6\;\mathrm{expression})\;+(-0.3559\;\times\;\mathrm{PPARGC}1\mathrm B\;\mathrm{expression})\;+\;(0.1154\;\times\;\mathrm{FADS}1\;\mathrm{expression})\;+(-0.2844\;\times\;\mathrm{CPT}1\mathrm B\;\mathrm{expression})\;+\;(0.1872\;\times\;\mathrm{HSD}17\mathrm B1\;\mathrm{expression})\;+(0.193\;\times\;\mathrm{OSBPL}10\;\mathrm{expression})\;+\;(0.1156\;\times\;\mathrm{AKR}1\mathrm B1\;\mathrm{expression})\;+\;(-0.2434\;\times\;\mathrm{CCDC}58\;\mathrm{expression})\;+(0.064\;\times\;\mathrm{PLA}2\mathrm G2\mathrm F\;\mathrm{expression})$$

**Fig. 5:**
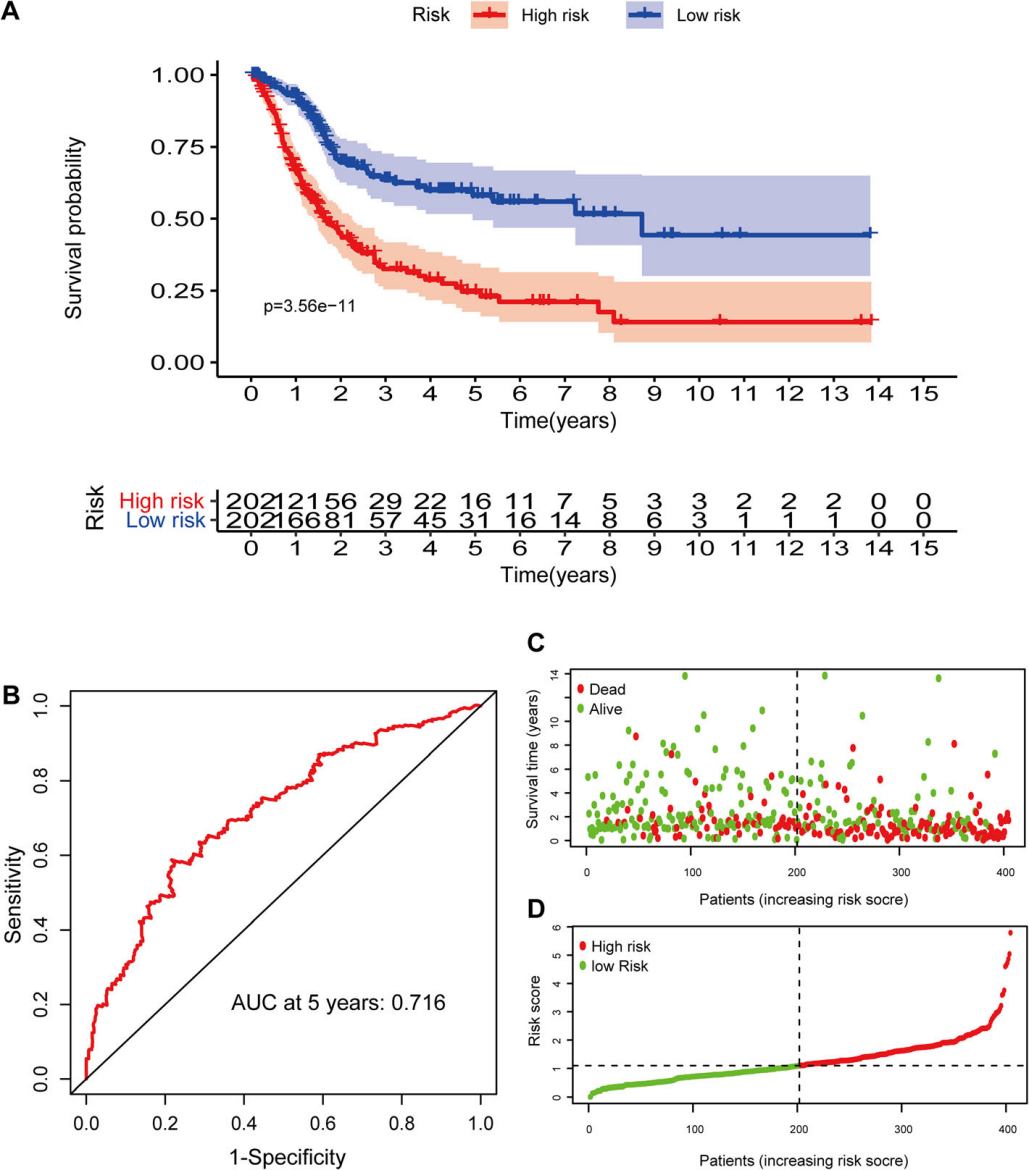
Construction of a prognostic signature based on 11 LRGs in TCGA set. **A** Kaplan-Meier survival analysis of BLCA patients between high-risk groups and low-risk groups. **B** Time-independent receiver operating characteristic (ROC) analysis of risk scores predicting the overall survival. **C** Distribution of LRG-based risk score and different patterns of survival status and survival time between the high- and low-risk groups

Patients were then divided into high- and low-risk groups on the basis of the median value. PCA and t-SNE analysis indicated distinct dimensions among different groups (Fig. 7A and C). Patients in the high-risk group showed poorer prognosis than those in the low-risk group (*P* < 0.05) (Fig. 5A and C). Time-dependent ROC analysis indicated that the prognostic accuracy of the 11 LRG signature in the TCGA set was 0.716 at 5 years (Fig. 5B).

### Validation of the LRGs signature in GEO dataset

**Fig. 6:**
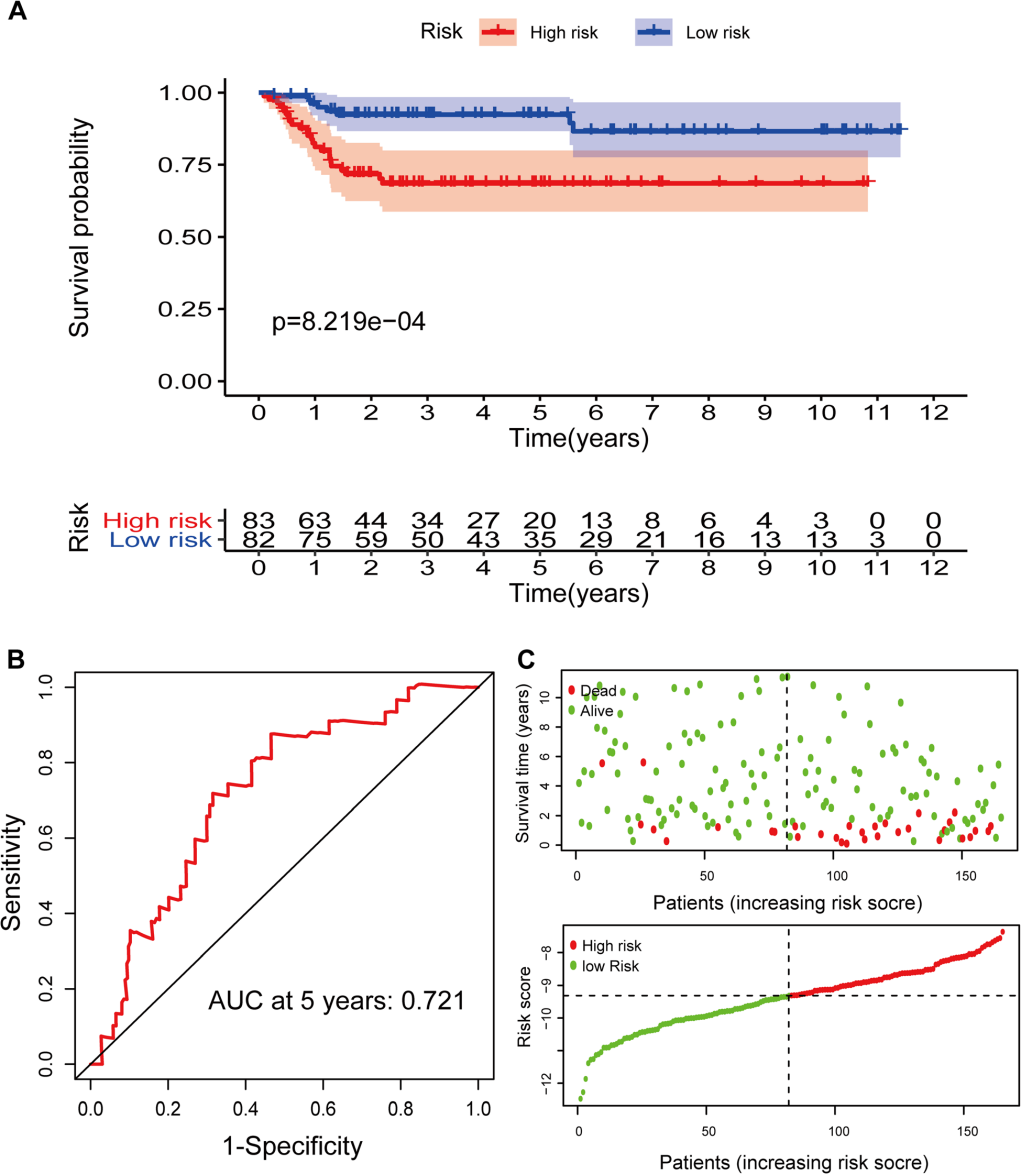
Validation of the prognostic signature based on 11 LRGs in GEO set. **A** Kaplan-Meier survival analysis of BLCA patients between high-risk groups and low-risk groups. **B** Time-independent receiver operating characteristic (ROC) analysis of risk scores predicting the overall survival. **C** Distribution of LRG-based risk score and different patterns of survival status and survival time between the high- and low-risk groups

**Fig. 7:**
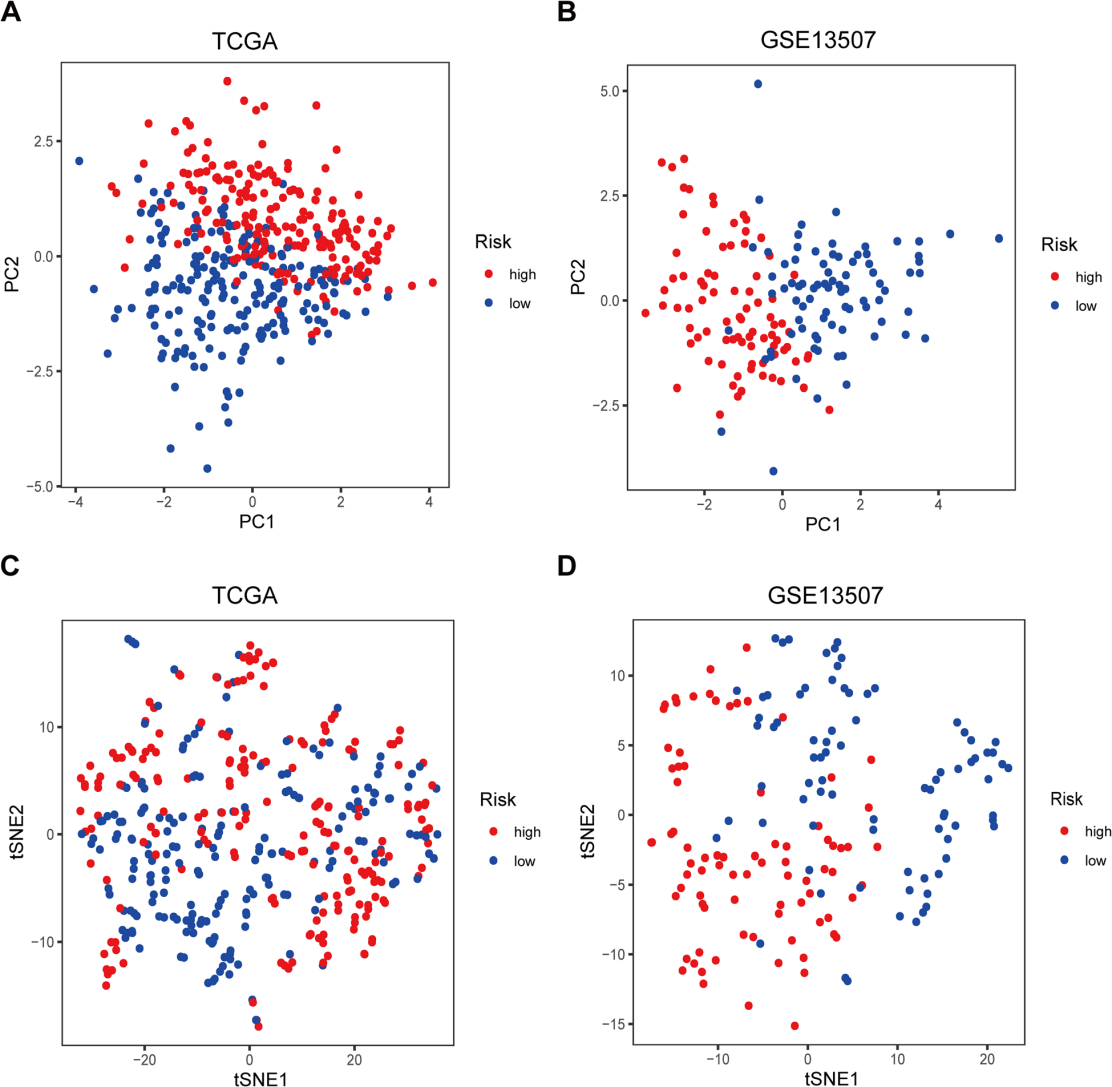
PCA and t-SNE analysis. **A** PCA analysis in the TCGA set. **B **PCA analysis in the GEO set. **C **t-SNE analysis in the TCGA set. **D **t-SNE analysis in the GEO set

To ensure the prediction value of the LRG signature, GSE13507 was served as a validation set to validate our results. According to the LRG-based classifier identified above, the BLCA patients in the validation sets were divided into a high- and a low‐risk group by the median risk score. In accord with the results above, significantly higher survival rates were observed in the low‐risk group than in the high‐risk group in the validation set (Fig. 6A and C). PCA and t-SNE analyses also indicated distinct dimensions among different groups (Fig. 7B and D). Time‐dependent ROC analysis indicated that the prognostic accuracy of the LRG signature was 0.721 at 5 years (Fig. 6B).

### The relationship between risk scores and clinical characteristics

**Fig. 8:**
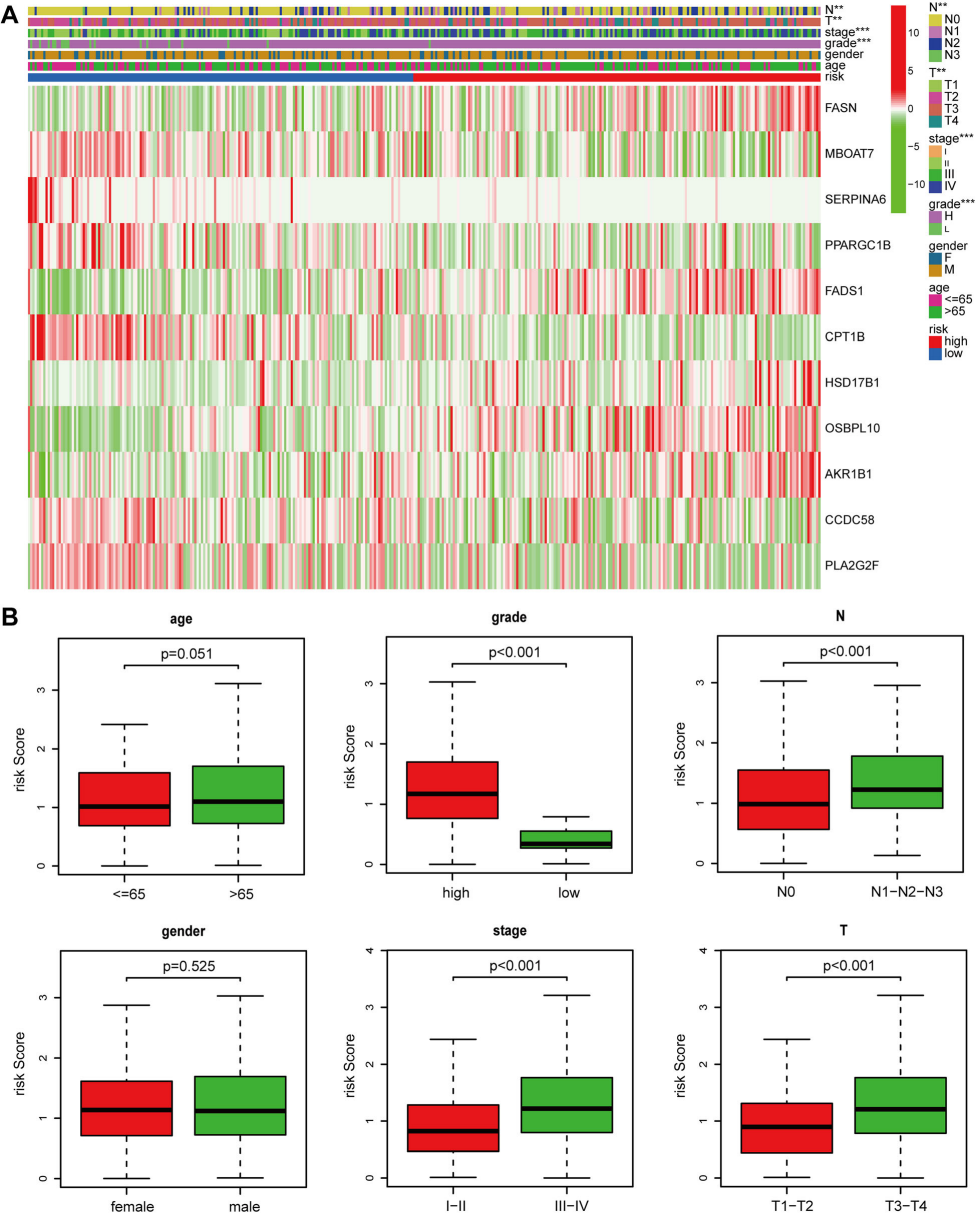
The correlation between the risk scores and clinicopathological factors. **A** Heatmap showed the correlation between the risk scores and clinicopathological factors. **B** Boxplot showed the correlation between the risk scores and clinicopathological factors

**Fig. 9:**
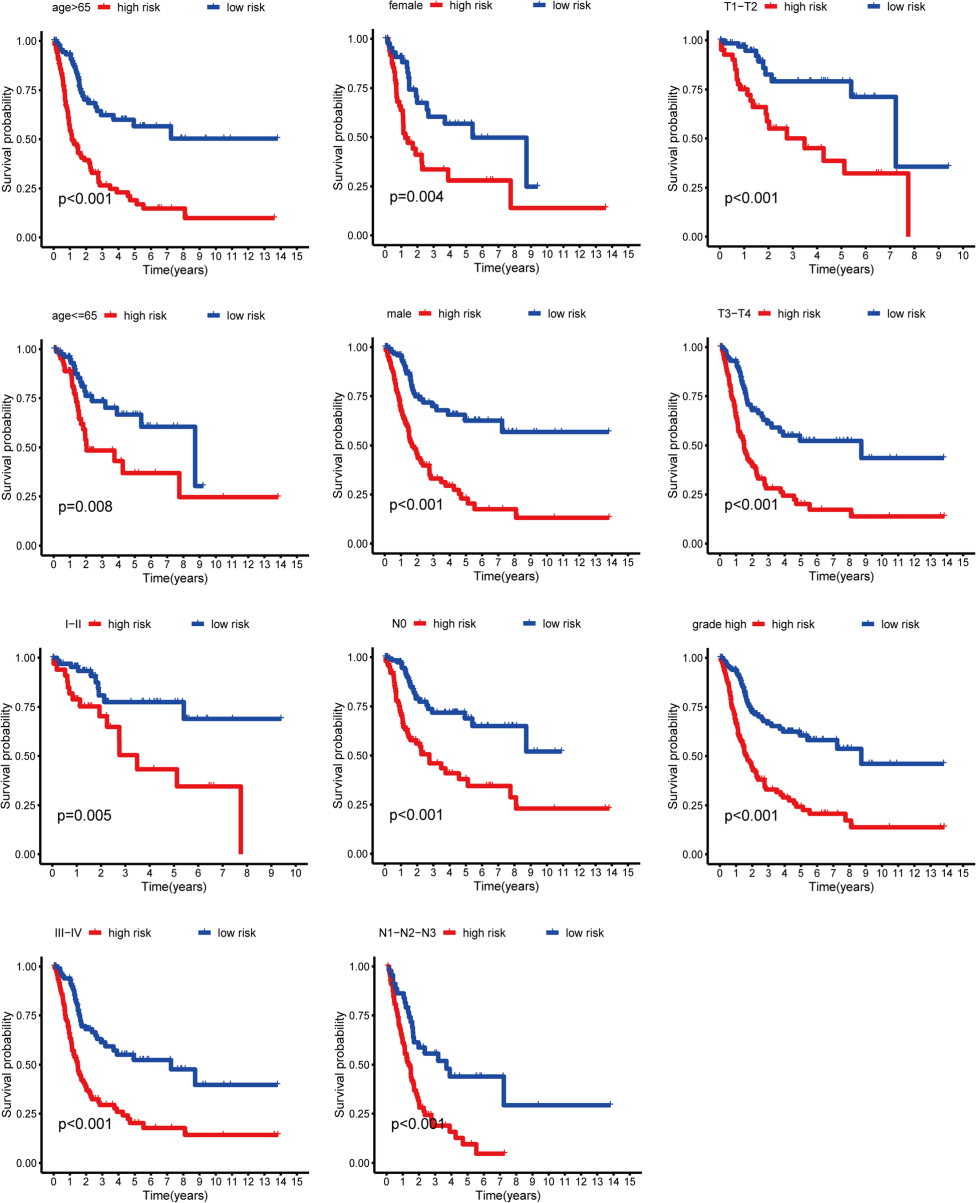
Kaplan-Meier curves of OS differences stratified by gender, age, T stage, N stage, tumor grade, or TNM stage between the high-and low-risk groups in the TCGA set

To further explore the correlation between risk scores and clinical characteristics, we analyzed the differences of the risk scores in the various subgroups stratified by clinical characteristics. The results indicated that the risk scores were closely related to clinical characteristics and significantly elevated in subgroups of high pathological grade, T stage (T3 and T4), N stage (N1-N2-N3), and TNM stage (Stage III and Stage IV) (Fig. 8A and B). However, the risk score in the age or gender subgroups was not statistically different. Subsequently, we further investigated the prognostic value of the LRG signature stratified by age (> 65 years or ≤ 65 years), TNM stage (I + II or III + IV), pathological grade (high), T stage (T1-T2 or T3- T4), gender (female or male), and N stage (N0 or N1-N2-N3). The Kaplan–Meier analysis suggested that patients with high risk scores had worse outcomes than those with low-risk scores in all the subgroups such as male or female, age (> 65 years) or ( < = 65 years), T stage (T1-T2) or (T3-T4), N stage (N0) or (N1-N2-N3), pathological grade (high) and TNM stage (Stage I–II) or (Stage III–IV) (all *P* < 0.05), these results suggested that risk scores might serve as an effective indicator for predicting the over survival of patients with BLCA (Fig. 9).

### Independent prognostic role of LRGs signature

**Fig. 10:**
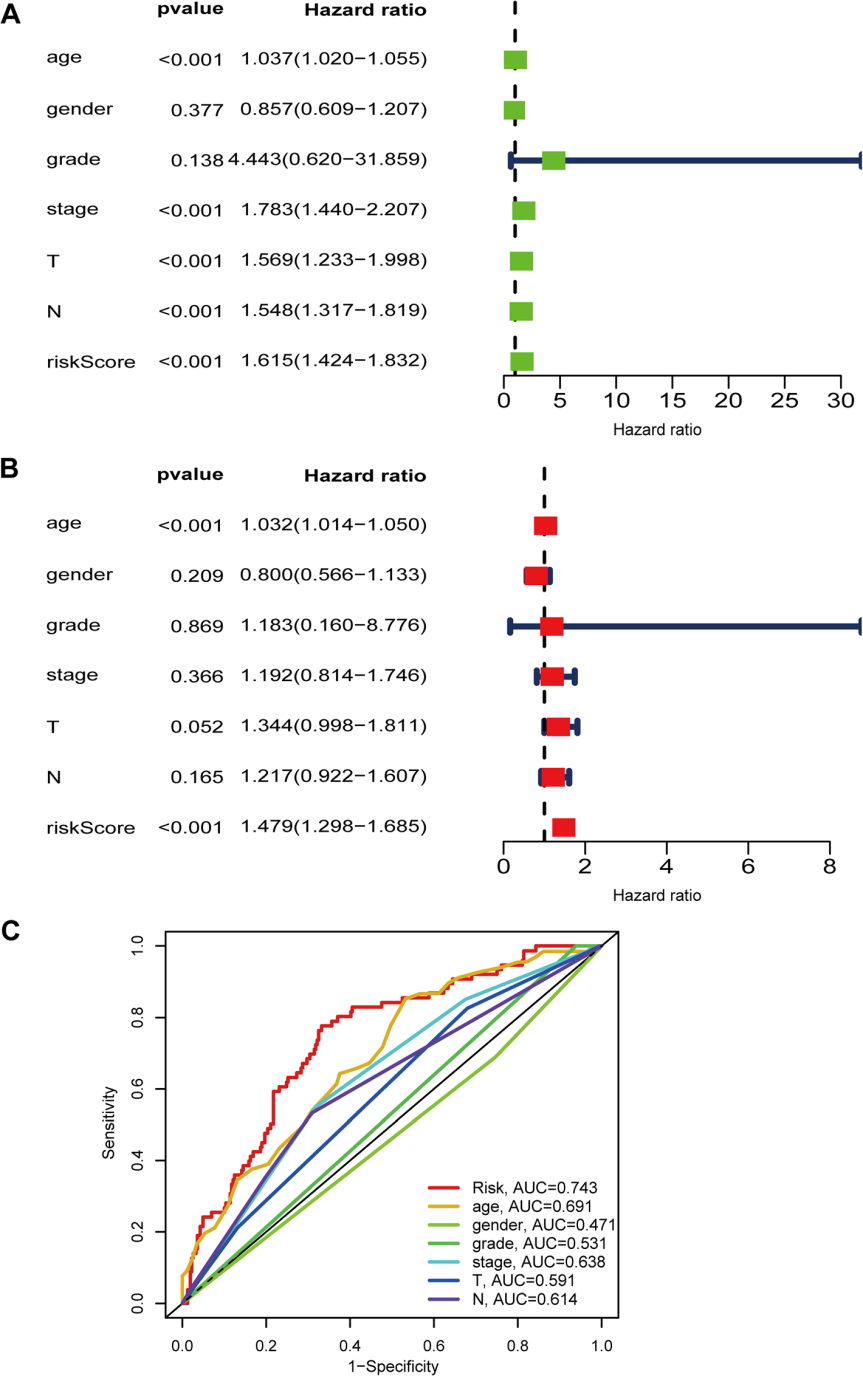
The risk signature was an independent prognostic factor for BLCA in TCGA set. **A **The correlations between the risk score for OS and clinicopathological factors by univariate Cox regression analysis. **B **The correlations between the risk score for OS and clinicopathological factors by multivariate Cox regression analysis. **C** ROC curves of the clinical characteristics and risk score

Univariate and multivariate Cox regression analyses were performed to explore the independence of the LRG signature by comparing the clinical features including gender, age, grade, TNM stage, T stage, and N stage. Age (HR = 1.037, 95 % CI = 1.020–1.055; *P* < 0.001), TNM stage (HR = 1.783, 95 % CI = 1.44–2.207, *P* < 0.001), T stage (HR = 1.569, 95 % CI = 1.233–1.819, *P* < 0.001), N stage (HR = 1.548, 95 % CI = 1.317–1.819, *P* < 0.001), and risk score (HR = 1.615, 95 % CI = 1.424–1.832, *P* < 0.001) were significantly associated with OS in the univariate analysis (Fig. 10A). Multivariate analysis suggested that age (HR = 1.032, 95 % CI = 1.014–1.05, *P* < 0.001) and risk score (HR = 1.479, 95 % CI = 1.298–1.685, *P* < 0.001) were also significantly associated with OS (Fig. 10B). Therefore, this result indicated that the risk score was an independent prognostic predictor. The result of multivariate ROC analysis showed that risk scores displayed a much more favorable performance in predicting OS than traditional pathological prognostic factors (Fig. 10C).

### Construction of a nomogram

**Fig. 11:**
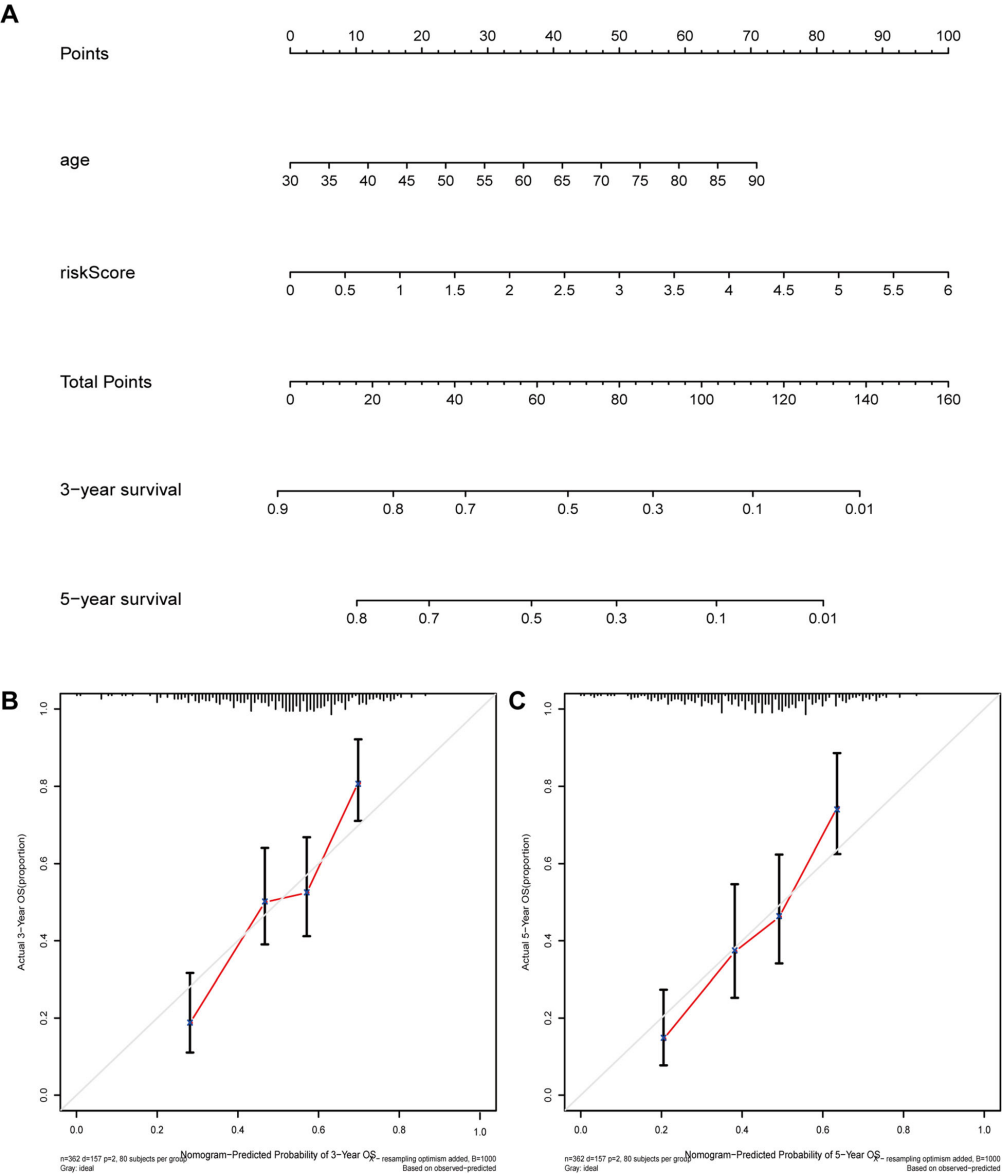
Construction of a nomogram. **A** Nomogram for predicting 3- or 5‐year OS. **B** The calibration plots for predicting 3-year OS. **C** The calibration plots for predicting 5-year OS

To better forecast the prognosis of BLCA patients, a nomogram consisting of the variables (age and risk scores) associated with OS was constructed (Fig. 11A). The calibration curve suggested that the nomogram showed good performance consistent with the nomogram’s 3- or 5‐year OS estimates and the Kaplan–Meier estimates (Fig. 11B and C).

### Analysis of immune cells infiltration

**Fig. 12:**
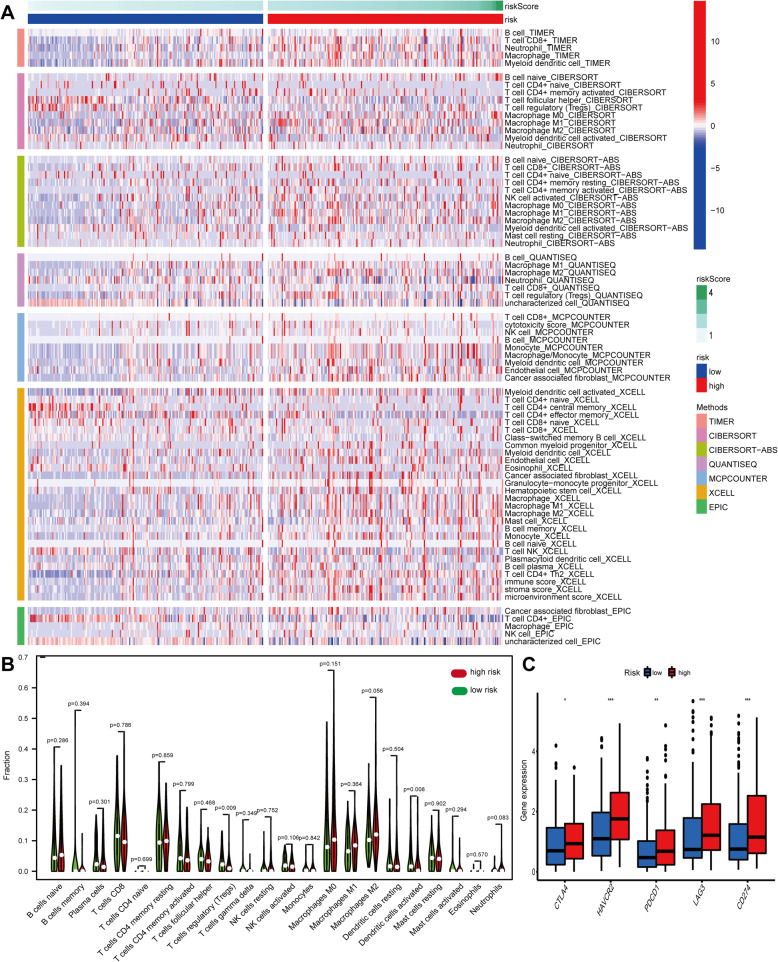
Immune cells infiltration between high-risk groups and low-risk groups. **A **Immune cells infiltration between different groups by CIBERSORT, QUANTISEQ, MCPcounter, XCELL, CIBERSORT-ABS, TIMER and EPIC algorithms. **B** CIBERSORT showed the correlation between different groups. **C** The expression of key immune checkpoint genes between different groups

The heatmap of immune responses based on CIBERSORT, QUANTISEQ, MCPcounter, XCELL, CIBERSORT-ABS, TIMER and EPIC algorithms is shown in Fig. 12A, which indicated that risk scores were correlated with immune cell infiltration in BLCA. The CIBERSORT results showed that risk scores were negatively related to the Treg cells and dendritic cell activation (Fig. 12B). In addition, the correlation between risk scores and key immune checkpoint genes (PDCD1, PD-L1, HAVCR2, LAG3 and CTLA4) was also explored. The results showed that the expressions of PDCD1, CTLA4, HAVCR2, PD-L1, and LAG3 were elevated in the high-risk groups (Fig. 12C), which suggested an immunosuppressive status in the high-risk groups.

### HPA database analysis

**Fig. 13:**
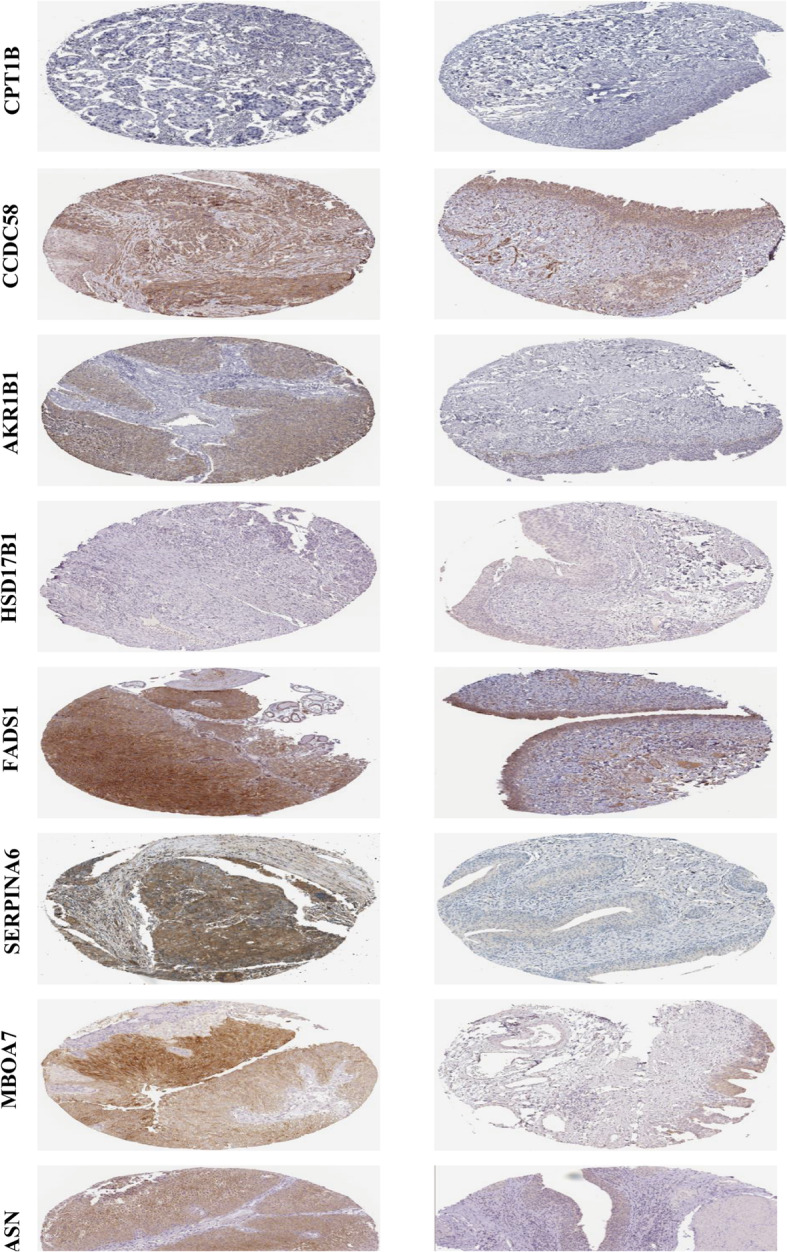
The protein expression of 11 prognostic genes by HPA database

HPA database was performed to explore the protein expression of 11 LRGs by assessing immunohistochemistry staining. HPA database has not included the protein expressions of OSBPL10, PLA2G2F, and PPARGC1B. Compared to the normal bladder tissues, the protein expressions of FASN, MBOA7, SERPINA6, FADS1, AKR1B1, and CCDC58 were obviously elevated in BLCA (Fig. 13). However, the protein expressions of HSD17B1 and CPT1B have no statistical criteria.

## Discussion

Significant advances in BLCA diagnosis and treatment have been achieved over the past two decades. However, the morbidity and mortality of BLCA remain unchanged owing to the aging of the population and absence of specific therapy. Consequently, novel prognostic biomarkers and treatments for BLCA need to be identified to improve patient prognosis. Accumulating studies have suggested that lipid metabolism dysregulation was involved in development and progression of various cancers, including lung cancer [18], prostate cancer [19], gastric carcinoma [20], and BLCA [13]. Despite the crucial role of lipid metabolism in BLCA, studies about the relationship between lipid metabolism and BLCA prognosis are rare.

In the present study, the potential mechanism and prognostic value of LRGs in BLCA were comprehensively investigated through bioinformatic analyses. 113 DELRGs were acquired by analyzing the expression of LRGs in BLCA tissues compared with normal bladder tissues in TCGA dataset. Then, PPI network and functional enrichment analyses were conducted to investigate the biological function of DELRGs in BLCA. GO and KEGG analyses showed that DELRGs were implicated in steroid metabolic process, fatty acid metabolic process, glycerophospholipid metabolism, PPAR signaling pathway, fatty acid metabolism, and AMPK signaling pathway. Finally, we utilized TCGA cohort to establish a prognostic risk signature and validated its high reliability and stability with GEO cohort. In addition, LRG-based signature was tightly related to inferior clinical characteristics, including TNM stage, T stage, N stage, and grade. Subgroups analyses also showed that patients with low risk scores had better outcomes than those with high risk scores. The results of univariate and multivariate analysis indicated that LRG-based signature was an independent prognostic factor in BLCA. Furthermore, multivariate ROC analysis also confirmed that risk scores were much more accurate in predicting OS than traditional pathological prognostic factors. Nomogram analysis indicated that the prognostic signature could be used to predict the outcomes of BLCA patients.

Increasing evidence has confirmed that immune cell infiltration was strongly associated with the development, progression and prognosis as well as the treatment of BLCA. In addition, metabolic remodeling could influence the functions of immune cell. Therefore, the relationship between the risk scores and immune cells infiltration was investigated. The results showed that Treg cells and dendritic cell activation has significantly elevated in the low-risk group compared with the high-risk group. Tregs could promote tumor progression by suppressing effective antitumor immunity and reduce immunotherapy benefits. Patients with BLCA in the high-risk groups had higher expression of PD1, CTLA4 PD-L1, HAVCR2 and LAG3 than those in the low-risk groups, suggesting that the unfavorable prognosis of patients in the high-risk groups might be partly due to the immunosuppressive environment and elevated expression of immune checkpoint genes. Furthermore, our results also suggested that patients in high-risk groups might benefit from immunotherapy.

Fatty acid desaturase 1 (FADS1), located on chromosome 11q12-11q13.1, is a member of the fatty acid desaturase gene family. Studies on FADS1 have primarily focused on its polymorphisms and deem FADS1 a rate-limiting enzyme in the biosynthesis of long-chain polyunsaturated fatty acid precursors of eicosanoids [21]. FADS1 has been reported to play a crucial role in many diseases, including type-1 diabetes and cancer [22, 23]. FADS1 can promote the progression of laryngeal squamous cell carcinoma by activating the AKT/mTOR signaling pathway [24]. Reduced FADS1 expression has been related to poor prognosis in NSCLC patients [22]. Jiao reported that FADS1 overexpression was positively correlated with tumor grade in BLCA [25]. Further study indicated that FADS1 knockdown inhibited BLCA cell proliferation by arresting the cell cycle. Membrane bound O-acyltransferase domain containing 7 (MBOAT7) has been reported to play an important role in inflammation [26]. Heinrichs found that MBOAT7 might contribute to GC susceptibility through inflammation [27]. MBOAT7 was overexpressed in renal cancer cells and high MBOAT7 expression was associated with poor prognosis in ccRCC [28].CCDC58 may play a tumor-promoting role in endometrial cancer. Aldo-keto reductase 1 member B1 (AKR1B1) could be involved in various signaling pathways, such as epithelial to mesenchymal transition (EMT) [29], inflammatory responses [30], and the mTOR pathway [31]. An increasing numbers of studies have suggested that AKR1B was involved in cancer progression [32]. Carnitine palmitoyltransferase 1B (CPT1B) has been reported to be correlated with tumor proliferation and metastasis by regulating EMT in BLCA [33]. Overexpression of CPT1B was correlated with worse OS in prostate cancer [34]. Further study revealed that AR-mediated CPT1B promoted castration-sensitive and castration‐resistant prostate cancer (CRPC) progression by upregulating AKT expression and phosphorylation. Peroxisome proliferator-activated receptor gamma coactivator 1 beta (PPARGC1B) could increase its transcriptional activity by selectively interacting with ERa, which plays a vital role in the ER signaling pathway [35]. PPARGC1B could inhibit miR-21 mediated fatty acid metabolism [36]. SERPINA6 is related to chemotherapy resistance in breast cancer [37].

Furthermore, CMAP dataset was used to identify five potential small molecule drugs highly related to LRGs for the treatment of BLCA patients. Flurbiprofen, a nonselective cyclooxygenase suppressor, is commonly used to control inflammation and pain during surgery. Recently, flurbiprofen has been reported to exert anticancer effects via suppressing proliferation and inducing apoptosis in several tumors [38, 39]. Meclizine could inhibit breast cancer cell clonogenesis in vitro [40]. Alfuzosin, an alpha1-adrenergic receptor antagonist, that is widely generally used for the treatment of hypertension and benign prostatic hyperplasia, reduced the growth of PC3 prostate tumor cells. Fenoprofen, a nonsteroidal anti-inflammatory drug, has been reported to be related to the incidence and metastases of prostate cancer [41]. Further research has shown that fenoprofen decreased the survival of prostate cancer cells by upregulating the expression of p75NTR (a neurotrophin receptor).

### Study strengths and limitations

The major strength of this study is the construction and validation of a prognostic signature based on lipid metabolism-related genes that is closely related to prognosis of BLCA patients. The main limitation of the study is the absence of experimental validation in vivo and vitro. Therefore, further experiments should be performed to validate the functions of lipid metabolism-related genes in BLCA.

## Conclusions

In conclusion, the expression, prognostic value, and function of lipid metabolism-related genes in BLCA were investigated by comprehensive bioinformatic analyses. A novel prognostic signature comprising 11 genes involved in lipid metabolism for predicting the outcomes of patients with BLCA was established and validated. In addition, the prognostic signature could serve as an indicator for predicting the therapeutic effect of immunotherapy in BLCA.

## Supplementary Information

**Additional file 1.**

## Data Availability

The raw data of this study are derived from the TCGA database (https://portal.gdc.cancer.gov/) and GEO data portal (https://www.ncbi.nlm.nih.gov/geo/), which are publicly available databases.

## Abbreviations

BLCA: Bladder cancer
LRGs: Lipid metabolism-related genes
MSigDB: Molecular Signature Database
DELRGs: Differentially expressed lipid metabolism-related genes
TCGA: The Cancer Genome Atlas
GEO: Gene Expression Omnibus
KEGG: Kyoto Encyclopedia of Genes and Genomes
FDR: False discovery rate
FC: Fold change
GO: Gene ontology
